# Assessment of Self-Stigma, Self-Esteem, and Asthma Control: A Preliminary Cross-Sectional Study Among Adult Asthmatic Patients in Selangor, Malaysia

**DOI:** 10.3389/fpubh.2019.00420

**Published:** 2020-01-22

**Authors:** Sohail Ahmad, Ahmad Izuanuddin Ismail, Mohd Arif Mohd Zim, Nahlah Elkudssiah Ismail

**Affiliations:** ^1^Faculty of Pharmacy, MAHSA University, Jenjarom, Malaysia; ^2^Respiratory Unit, Faculty of Medicine, Universiti Teknologi MARA, Batu Caves, Malaysia

**Keywords:** stigma, self-esteem, asthma, control, factors, Malaysia

## Abstract

**Purpose:** The elusive goal of asthma management guidelines is to achieve and maintain good asthma control in asthmatic patients. Against a background of long-term respiratory limitations when living with asthma, stigma and low self-esteem have also been identified as the social phenomenon among adult asthmatics. This study aimed to assess the levels of self-stigma, self-esteem, and asthma control, and to investigate the impact of self-stigma and self-esteem as psychosocial factors on asthma control in Malaysian adults living with asthma.

**Materials and Methods:** In this multicenter cross-sectional study, post-ethics approval and patients' consents, 152 stable asthmatic patients (aged > 18 years old; nil cognitive disability; not diagnosed with other respiratory diseases) were recruited from four respiratory clinics in Selangor, Malaysia. The patients' socio-demographic, medical, and psychosocial (self-stigma and self-esteem) data were recorded in a pre-validated, self-designed questionnaire. All data were analyzed descriptively and inferentially (independent *t*-test/one-way ANOVA, and multiple linear regression) using the Statistical Package for Social Sciences (SPSS®).

**Results:** The enrolled patients showed moderate levels of self-stigma (62.12 ± 6.44) and self-esteem (29.31 ± 3.29), and not well-controlled asthma (17.58 ± 3.99). The number of patients' visits to emergency rooms because of asthma [CI (−1.199, 0.317), *p* < 0.001] was the significant predictor to asthma control among all selected study variables from socio-demographic and medical data. Moreover, from psychosocial factors both self-stigma [CI (−0.367, 0.190), *p* < 0.001], and self-esteem [CI (−0.007, 0.033), *p* = 0.041] found to be the significant predictors of asthma control.

**Conclusion:** The preliminary evidences presented in this study found that frequent emergency room visits, high self-stigma and low self-esteem in asthma patients becomes more apparent with poor asthma control. Educational interventions to reduce patients' self-stigma and improve self-esteem are needed to achieve optimal control of asthma.

## Introduction

High prevalence and poor control of asthma make asthma management a major public health issue worldwide (1). There is no cure of asthma; therefore, the focus of asthma self-management strategies and programs, is to help the asthma patients by controlling the disease, preventing the worsening symptoms and minimizing the severity of asthma (2, 3). Despite the availability of regularly updated asthma management guidelines and effective asthma treatment, control of asthma in the majority of patients is still in suboptimal situation (4).

Asthma has diverse psychosocial implications that may affect the self-management of asthma (5). Against a background of long-term respiratory limitations when living with asthma, stigma and low self-esteem have also been identified as a social phenomenon among adult asthmatics (6). Self-stigma and self-esteem of asthmatic patients may influence the asthma control either directly or indirectly. The possible negative consequences because of self-stigma of asthma can be explained in terms of decreased self-efficacy in the management of asthma as well as the barriers it places on patients' access to healthcare and social relationships. Furthermore, these unfortunate implications may lead to the increased morbidity and a reduced quality of life of the asthmatics (7). The self-stigma of asthma may also result into medication non-compliance, patients' anxieties, poor control of symptoms of asthma, avoidance of inhaler use in public and inability to participate in the community actively (8). Similarly, self-esteem can influence the quality of life, physical and mental well-being of the patients living with chronic diseases including asthma (9).

Research has explored and identified the internalized stigma and low self-esteem in individuals living with asthma (2, 9), but less has been done to evaluate the levels of self-stigma and self-esteem for the respective impact on asthma control. Moreover, at present there is only limited information available on the extent to which socio-demographic, medical and psychosocial (self-stigma, self-esteem) factors influence asthma control. Therefore, this study aimed to evaluate the level of self-stigma, self-esteem and asthma control in adult asthma patients and investigate the impact self-stigma and self-esteem as psychosocial factors on asthma control in Malaysian adults living with asthma.

## Materials and Methods

### Ethics Statement

**Figure 1:**
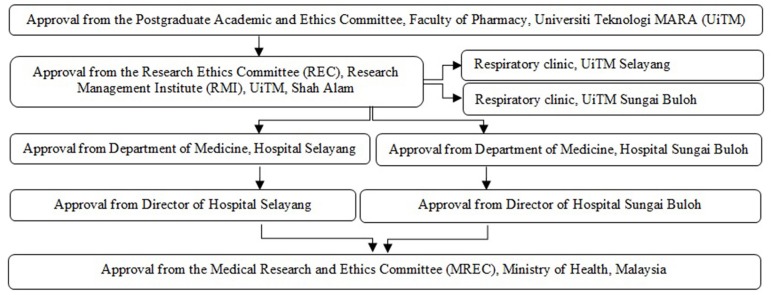
Ethical approval procedure.

This study protocol was approved by the Postgraduate Academic and Ethics Committee (600-FF-(PT-9/19)) and ethics approval was obtained from the Institutional Research Ethics Committee (IREC) (600-RMI-(5/1/16)) and Medical Research and Ethics Committee (MREC), Ministry of Health (MOH), Malaysia, through the National Medical Research Registry (NMRR-14-557-20184). The complete procedure of ethical approval is depicted in Figure 1.

### Study Design, Population, and Sample Size Calculation

A cross-sectional study using a validated self-administered questionnaire was conducted among adult asthmatic patients attending four respiratory specialist clinics in Selangor, Malaysia. Only those asthmatic patients who met the inclusion criteria (stable asthmatic patients as specified by GINA, aged ≥ 18 years old, good understanding of Malay language) were recruited. Both genders were considered in this study. This study excluded those who had cancer of any organ at any stage, congestive heart failure, or strokes. Patients diagnosed with psychiatric illnesses and/or taking associated medication (such as for mood disorder or severe depression), and patients with hearing and speech impairments were also excluded from this study.

The sample size of this study was calculated by using estimated number of patients as obtained by the hospital records and by assuming 50% response distribution, 95% confidence interval with 5% margin of error. The minimum recommended sample size was 132 patients by Raosoft® online sample size calculator. Twenty additional patients were enrolled to overcome erroneous results and to increase the reliability of the conclusion (10). The subjects were selected using purposive sampling method. This sampling technique was adopted for two reasons; first, by this method only those patients were targeted that met the defined inclusion criteria. Secondly, this method made the patient recruitment process more systematic. Before approaching the patients, the list of scheduled appointments was gone through in the early morning of the clinic day and subjects of interest were identified.

### Questionnaire (Study Instrument)

The questionnaire was adapted from previous studies and modified to suit the Malaysian population. The questionnaire comprised four main parts. In part A, patients' socio-demographic (gender, age, ethnicity, educational level, occupation, income) and medical data (number of years since diagnosed as asthmatic, emergency visits, hospitalization and intubation, triggering factors for asthma, asthma treatment (own medication and devices for asthma), body mass index (BMI) and medical data were recorded. In part B, the control of asthma was assessed by using the Malay version of the asthma control test (ACT). The ACT assessed the limitation of the activity, shortness of breath, night-time symptoms, intake of rescue medication, and patients' overall rating of their asthma control over the past 4 weeks (11). Part C of the questionnaire assessed the degree of self-stigma in asthmatic adults by using 22 items that were adapted from a Stigma Scale for Mental Health (12). Each item was scored on a 5-point Likert scale, where patients' responses varied from strongly agree to strongly disagree. Lastly, in part D of the questionnaire, a Self-Esteem Scale was used to determine the self-esteem of the adult asthmatic patients (13). This scale contained 10 items, and the responses of patients were recorded on a 4-point Likert scale with the given statements, ranging from strongly agree to strongly disagree. There were 5 reverse-coded items (item numbers: 2, 5, 6, 8, 9) in part D.

### Translation of Questionnaire

The questionnaire was designed into two versions: English and Malay. The English version was developed first and then translated into Malay by forward-backward-forward translation technique. The translation process was taken by bilingual experts. After harmonizing the translated versions, pre-test cognitive debriefing of the instrument was done to actively test the feasibility, interpretation, understanding, and cultural relevance among eight adult asthma patients. Lastly, the finalized Malay version was ready to administer for qualitative validation and pilot testing. The content validation of finalized version was done by a senior panel comprising of three practicing respiratory physicians, two senior pharmacists and one expert in questionnaire validation. Face validation was done by administering Malay version of questionnaire in 10 asthma patients to ensure that the statements convey the same meanings to the patients as the investigator intended. Modifications were made based on the outcomes of content and face validation feedbacks. A pilot study was conducted with 30 subjects enrolled from the study settings and the initial validation findings were published (3).

### Data Collection

On the start of each respiratory clinic day, the list of patients who had the scheduled appointments was acquired by the nurse on duty. After identifying the potential participants who met the inclusion criteria, patients were asked for their voluntary participation and consent after the consultation with their respiratory physicians. The patients were given ample stationery and time in completing the instrument that on average took 15 min to complete. Prior to take patients consent to participate in this study, they were instructed briefly about the study using subject information sheet. Furthermore, patients' socio-demographics, medical data and asthma control were recorded from electronic medical records. The patients were encouraged to complete every question in chronological order without skipping any item of self-stigma and self-esteem scales. Some patients had previous experience in participating research including questionnaire-based studies and clinical trials that made them feel at ease in agreeing and completing the present study. Once patients completed the questionnaire, the completeness of the questionnaire was checked in terms of missing responses. In case of incomplete response, patients were requested to complete the missing information. Privacy and confidentiality of information was guaranteed.

### Data Analysis

After completing the data collection process, editing of the raw data was carried out to ensure the completeness of the data. Editing involved checking the data collection forms for omissions, and legibility. The raw data were manually entered into a data file. The pre-coding method was used whereby all categorical items were pre-coded with numerical values; for instance, each trigger of asthma was coded 1 for Yes and 0 for No. For ACT, the questions were scored from 1 (worst) to 5 (best). Higher score of ACT reflected better control of asthma. The asthma was categorized as poorly controlled (score = 5–15), not well-controlled (score = 16–20) and well-controlled asthma (score = 21–25). Moreover, items were scored on a 5-point Likert scale ranging from strongly agree (score = 5) to strongly disagree (score = 1) for self-stigma. The scores were summed to provide an overall stigma score. Higher score reflected high stigma. The patient's stigma score of 75% or above (score ≥ 83/110) reflected severe stigma, 50–74% (score = 55–82/110) represented moderate and 49% or below (score < 55/110) reflected mild stigma. For self-esteem, patients responses were coded on a 4-point Likert scale from strongly agree (score = 4) to strongly disagree (score = 1). Five items in the scale were reverse coded (item number: 2, 5, 6, 8, 9) i.e., for these items scoring was reversed from strongly agree (score = 1) to strongly disagree (score = 4). Higher score reflected the greater self-esteem. Total score of 75% or above (score ≥ 30/40) reflected high self-esteem, 50–74% (score = 20–29/40) represented moderate and 49% or below (score < 20/40) reflected low self-esteem.

All data were analyzed using the Statistical Package for Social Sciences (SPSS®), version 21. The independent variables from socio-demographical and medical data were analyzed descriptively. The influence of the socio-demographic and medical characteristics on self-stigma, self-esteem and asthma control was tested using independent *t*-test and one-way ANOVA where appropriate. Moreover, Pearson product-moment correlation coefficients were computed to assess the correlations between the dependent and independent study variables. Only continuous study variables were included in the correlation analyses and these correlations were categorized into weak (*r* = 0.10–0.29), moderate (*r* = 0.30–0.49) and strong (*r* = 0.5–1) correlations as suggested by Cohen et al. (14). In addition, multiple linear regression models were developed to predict the impact of variables from socio-demographic and medical data, and psychosocial factors (self-stigma, self-esteem) on asthma control. The level of statistical significance was set a priori at *p* < 0.05 for all statistical tests.

## Results

The response rate of the enrolled asthma patients was 85.8%. The majority of the respondents were female (*n* = 107; 70.4%), Malay (*n* = 81; 53.3%) and housewives (*n* = 50; 32.9%). The mean ± standard deviation (SD) age of the respondents and number of years since diagnosed as asthmatic were 52.03 (±15.11), and 21 (±15.66) years, respectively. More than two third of the respondents (74.6%) completed at least secondary education. Mostly patients either completed their college (*n* = 43, 28.3%) or secondary school education (*n* = 46, 30.3%). The details of patients' socio-demographic and medical data are shown in Tables 1, 2, respectively.

**Table 1:** Socio-demographic data of enrolled asthmatic patients (*n* = 152).

**Sr. #**	**Items**	**Category**	**Statistics**
1	Gender, *n* (%)	Male	45 (29.6)
		Female	107 (70.4)
2	Age (years old), mean (± *SD*)		52.03 (15.11)
3	Ethnicity, *n* (%)	Malay	81 (53.3)
		Chinese	30 (19.7)
		Indian	41 (27.0)
		Others	0 (0)
4	Highest completed educational level, *n* (%)	Primary school	40 (26.3)
		College/Polytechnic	43 (28.3)
		Secondary school	46 (30.3)
		University	22 (14.5)
		Others	1 (0.7)
5	Occupation, *n* (%)	Student	10 (6.6)
		Professional	25 (16.4)
		Housewife	50 (32.9)
		Unemployed	14 (9.2)
		Non-professional	17 (11.2)
		Retired	23 (15.1)
		Self employed	12 (7.9)
		Other	1 (0.7)
6	Income per household/month (RM), *n* (%)	<1,000	51 (33.6)
		1,000–2,000	59 (38.8)
		2,001–3,000	23 (15.1)
		>3,000	19 (12.5)
7	Number of years since diagnosed as asthmatic (years), mean (± *SD*)		21 (15.66)

*Sr, Serial*.

**Table 2:** Medical data of enrolled asthmatic patients (*n* = 152).

**Sr. #**	**Items**	**Category**		**Statistics**
1	Emergency visits for asthma, *n* (%)	Times/last year		
			Yes	73 (48.1)
			No	79 (51.9)
2	Hospitalized for asthma, *n* (%)	Times/last year		
			Yes	34 (22.3)
			No	118 (77.6)
3	Incubated for asthma, *n* (%)	Times/last year		
			Yes	3 (1.9)
			No	149 (98.1)
4	Medical Record, mean (± *SD*)	Weight (Kg)		68.60 (17.08)
		Height (m)		1.58 (1.02)
		BMI (Kg/m^2^)		33.44 (10.41)
5	Other disease, *n* (%)	Hypertension		16 (10.5)
		Diabetes		25 (16.4)
		Atopic eczema		9 (5.9)
		Other		17 (11.2)
		No other disease except asthma		85 (55.9)
6	Triggering factors *n* (%)	Air pollution	Yes	91 (59.9)
			No	61 (40.1)
		Dust	Yes	105 (69.1)
			No	47 (30.9)
		Respiratory infection	Yes	51 (33.6)
			No	101 (66.4)
		Chemicals	Yes	40 (26.3)
			No	112 (73.7)
		Exercise	Yes	41 (26.9)
			No	111 (73.1)
		Stress	Yes	60 (39.5)
			No	92 (60.5)
		Cigarette smoke	Yes	63 (41.4)
			No	89 (58.6)
		Furred animal	Yes	51 (33.6)
			No	101(66.4)
		Weather	Yes	106 (69.7)
			No	46 (30.3)
		Diet	Yes	51 (33.6)
			No	101 (66.4)
		Drugs	Yes	12 (7.9)
			No	140 (92.1)
		Others	Yes	3 (2)
			No	149 (98)

*Sr, Serial*.

From the results obtained, the overall mean (±SD) score of asthma control was 17.58 ± 3.99; reflecting not well-controlled asthma (mode: 19.0; median: 18.0; interquartile range (IQR): 6; minimum: 8; maximum: 25; range: 17). Moreover, the mean (±SD) scores of self-stigma: 62.12 ± 6.44 (mode: 58; median: 60; IQR: 8; minimum: 49; maximum: 76; range: 27) and self-esteem: 29.31 ± 3.29 (mode: 29; median: 29; IQR: 4; minimum: 19; maximum: 38; range: 19) suggested that they had moderate levels of self-stigma and self-esteem. The data of dependent variables was normally distributed; therefore, the independent *t*-test and one-way ANOVA (parametric tests) were used, where applicable. The findings of independent *t*-test/one-way ANOVA are shown in Tables 3, 4.

**Table 3:** Comparison of self-stigma, self-esteem, and asthma control across socio-demographic variables.

**Sr. #**	**Variables**	**Groups**	***N***	**Mean (± *SD*)**	***t*-stat (df)^**a**^/f-stat (df)^**b**^**	***p*-value**
**1**	**Age (years old)**					
	Self-stigma	18–35	29	61.90 (5.53)	0.291 (2)^b^	0.748
		36–45	18	61.17 (3.89)		
		>45	105	62.36 (6.44)		
	Self-esteem	18–35	29	29.14 (4.02)	0.113 (2)^b^	0.893
		36–45	18	29.61 (3.86)		
		>45	105	29.30 (2.99)		
	Asthma control	18–35	29	18.66 (3.33)	3.24 (2)^b^	0.042^*^
		36–45	18	19.00 (3.62)		
		>45	105	17.03 (4.13)		
**2**	**Gender**					
	Self-stigma	Male	45	63.07 (5.93)	1.17 (150)^a^	0.244
		Female	107	61.73 (6.64)		
	Self-esteem	Male	45	29.71 (3.46)	0.974 (150)^a^	0.332
		Female	107	29.14 (3.23)		
	Asthma control	Male	45	18.09 (4.33)	1.020 (150)^a^	0.310
		Female	107	17.37 (3.85)		
**3**	**Ethnicity**					
	Self-stigma	Malay	81	61.62 (6.36)	0.633 (2)^b^	0.532
		Chinese	30	63.10 (7.01)		
		Indian	41	62.41 (6.25)		
		Other				
	Self-esteem	Malay	81	29.11 (3.21)	2.59 (2)^b^	0.078
		Chinese	30	30.50 (3.35)		
		Indian	41	28.83 (3.33)		
		Others				
	Asthma control	Malay	81	17.27 (3.93)	0.628 (2)^b^	0.535
		Chinese	30	18.20 (4.00)		
		Indian	41	17.73 (4.17)		
		Others				
**4**	**Highest completed educational level**					
	Self-stigma	Primary school	40	60.85 (6.75)	1.542 (4)^b^	0.193
		College	43	61.95 (6.41)		
		Secondary school	46	62.50 (6.31)		
		University	22	63.45 (5.86)		
		Others	1	74		
	Self-esteem	Primary school	40	29.58 (2.98)	1.287 (4)^b^	0.278
		College	43	29.47 (3.56)		
		Secondary school	46	28.59 (3.09)		
		University	22	29.82 (3.63)		
		Others	1	34.00		
	Asthma control	Primary school	40	17.20 (3.71)	1.434 (4)^b^	0.226
		College	43	17.79 (3.85)		
		Secondary school	46	16.98 (4.29)		
		University	22	19.22 (4.02)		
		Others	1	15.00		
**5**	**Occupation**					
	Self-stigma	Student	10	62.40 (6.64)	1.372 (7)^b^	0.222
		Professional	25	60.08 (5.21)		
		Housewife	50	63.48 (7.74)		
		Unemployed	14	58.79 (5.45)		
		Non-professional	17	62.53 (6.39)		
		Retired	23	63.04 (6.24)		
		Self-employed	12	61.75 (4.22)		
		Other	1	66.00		
	Self-esteem	Student	10	28.00 (4.39)	1.176 (7)^b^	0.320
		Professional	25	29.84 (4.21)		
		Housewife	50	29.46 (2.88)		
		Unemployed	14	29.07 (2.02)		
		Non-professional	17	27.88 (1.94)		
		Retired	23	29.39 (1.62)		
		Self-employed	12	30.83 (1.58)		
		Other	1	29.00		
	Asthma control	Student	10	17.50 (4.19)	0.714 (7)^b^	0.660
		Professional	25	17.96 (4.37)		
		Housewife	50	17.10 (4.06)		
		Unemployed	14	18.64 (3.62)		
		Non-professional	17	16.29 (3.51)		
		Retired	23	18.17 (3.99)		
		Self-employed	12	18.00 (4.11)		
		Other	1	21.00		
**6**	**Income per household**/**month (RM)**					
	Self-stigma	<1000	50	63.50 (7.34)	1.369 (3)^b^	0.255
		1,000–2,000	60	61.08 (6.14)		
		2,000–3,000	23	62.39 (5.85)		
		>3,000	19	61.47 (5.22)		
	Self-esteem	<1,000	50	28.94 (2.76)	0.471 (3)^b^	0.703
		1,000–2,000	60	29.52 (3.22)		
		2,000–3,000	23	29.13 (4.53)		
		>3,000	19	29.84 (3.23)		
	Asthma control	<1,000	50	17.08 (4.21)	0.492 (3)^b^	0.689
		1,000–2,000	60	17.63 (3.67)		
		2,000–3,000	23	18.08 (4.25)		
		>3,000	19	18.10 (4.28)		

a independent t-test;

b one-way ANOVA;

* *p < 0.05. Sr, Serial*.

**Table 4:** Comparison of self-stigma, self-esteem and asthma control across medical data categories.

**Sr. #**	**Variables**	**Groups**	***N***	**Mean (± *SD*)**	**t-stat (df)^**a**^/f-stat (df)^**b**^**	***p*-value**
**1**	**Number of years since diagnosed as asthmatic**					
	Self-stigma	<5 years	27	60.63 (5.69)	2.413 (2)^b^	0.093
		5–10 years	27	60.56 (6.53)		
		>10 years	98	62.96 (6.52)		
	Self-esteem	<5 years	27	30.22 (3.25)	1.701 (2)^b^	0.186
		5–10 years	27	28.59 (3.86)		
		>10 years	98	29.25 (3.12)		
	Asthma control	<5 years	27	18.96 (3.67)	2.077 (2)^b^	0.129
		5–10 years	27	17.56 (3.58)		
		>10 years	98	17.20 (4.14)		
**2**	**Number of medicines**					
	Self-stigma	1 or 2	44	61.02 (4.96)	1.547 (110.3)^a^	0.125
		More than 2	108	62.57 (6.92)		
	Self-esteem	1 or 2	44	29.15 (3.59)	0.357 (150)^a^	0.721
		More than 2	108	29.37 (3.18)		
	Asthma control	1 or 2	44	17.52 (3.92)	0.110 (150)^a^	0.912
		More than 2	108	17.60 (4.04)		
**3**	**Number of triggers of asthma**					
	Self-stigma	<3	75	61.65 (5.35)	2.603^b^	0.077
		4–6	40	64.05 (6.79)		
		>7	37	61.00 (7.71)		
	Self-esteem	<3	75	29.34 (3.31)	0.009^b^	0.991
		4–6	40	29.27 (3.25)		
		>7	37	29.27 (3.40)		
	Asthma control	<3	75	18.42 (3.47)	3.433 ^b^	0.035*
		4–6	40	16.75 (4.42)		
		>7	37	16.75 (4.40)		
**4**	**Hospitalization**					
	Self-stigma	No	124	61.62 (5.93)	1.69 (33.81)^a^	0.101
		Yes	28	64.35 (8.07)		
	Self-esteem	No	124	29.37 (3.31)	0.485 (150)^a^	0.629
		Yes	28	29.03 (3.31)		
	Asthma control	No	124	18.00 (3.91)	2.849 (150)^a^	0.005*
		Yes	28	15.67 (3.85)		
**5**	**Emergency visits**					
	Self-stigma	No	75	61.84 (6.43)	0.537 (150)^a^	0.592
		Yes	77	62.40 (6.48)		
	Self-esteem	No	75	29.54 (3.27)	0.875 (150)^a^	0.383
		Yes	77	29.07 (3.32)		
	Asthma control	No	75	18.60 (3.81)	3.201 (150)^a^	0.002*
		Yes	77	16.58 (3.94)		
**6**	**BMI (kg/m**^**2**^**)**					
	Self-stigma	Underweight	11	61.72 (7.76)	3.943 (3)^b^	0.010*
		Normal	39	59.82 (4.70)		
		Overweight	55	64.23 (7.06)		
		Obese	47	61.65 (6.02)		
	Self-esteem	Underweight	11	28.90 (5.16)	0.185 (3)^b^	0.906
		Normal	39	29.61 (3.05)		
		Overweight	55	29.27 (3.31)		
		Obese	47	29.19 (3.02)		
	Asthma control	Underweight	11	16.09 (3.26)	4.763 (3)^b^	0.003*
		Normal	39	19.38 (3.27)		
		Overweight	55	16.52 (4.51)		
		Obese	47	17.65 (3.58)		
**7**	**Co-morbidity**					
	Self-stigma	No	75	61.94 (6.40)	0.236 (124)^a^	0.814
		Yes	51	62.21 (6.07)		
	Self-esteem	No	75	29.16 (3.37)	0.125 (124)^a^	0.901
		Yes	51	29.23 (3.27)		
	Asthma control	No	75	17.58 (3.96)	0.380 (124)^a^	0.705
		Yes	51	17.86 (4.05)		

a independent t-test;

b one-way ANOVA;

* *p < 0.05. Sr, Serial*.

For both self-stigma and self-esteem, none of the variable categories showed any significant differences from all study variables except self-stigma score across BMI groups. For asthma control statistically significant differences were observed among age categories from socio-demographic data; whereas, the frequency of hospitalizations and emergency visits, number of asthma triggers, and BMI from medical data.

As shown in Table 5, asthma control showed moderate negative correlation with number of emergency visits and weak negative correlation with number of hospitalizations and number of asthma triggers from medical data. Self-stigma showed weak positive correlation with emergency visits. Asthma control showed moderate negative and weak positive correlations with self-stigma and self-esteem, respectively. All remaining correlations were statistically insignificant.

**Table 5:** Correlation matrix of asthma control, self-stigma and self-esteem with selected study variables.

**Study variables**	**Asthma control**		**Self-stigma**		**Self-esteem**	
	**r**	***p***	**r**	***p***	**r**	***p***
Age (years)	−0.107	0.191	0.136	0.951	0.103	0.208
Number of years diagnosed	−0.119	0.145	0.146	0.072	−0.070	0.393
Number of hospitalizations	−0.197	0.015^*^	0.145	0.075	−0.10	0.907
Number of emergency visits	−0.316	< 0.001**	0.217	0.007^*^	−0.061	0.457
Number of intubations	−0.101	0.217	0.032	0.695	0.014	0.867
Number of triggers	−0.186	0.022	0.033	0.690	−0.063	0.440
BMI	−0.026	0.061	0.139	0.087	0.008	0.558
Self-stigma	−0.456	< 0.001**			−0.148	0.069
Self-esteem	0.206	0.008				

*r, Pearson correlation coefficient*.

** *p < 0.01*.

* *p < 0.05*.

Multiple linear regression analysis was used to develop a model for predicting level of asthma control from gender, patient's age, ethnicity, education level, occupation, monthly income per household (RM), number of years diagnosed as asthmatic, emergency visits and hospitalization due to asthma during past year. Table 6 shows that the number of patients' visits to emergency department because of asthma [CI (−1.199, to 0.317), *p* < 0.001] was the significant predictor to asthma control among all selected study variables from socio-demographic and medical data. The overall model explains 12.9% of the variance [*R*^2^ = 0.129, *F*_(9, 142)_ = 2.343, *p* = 0.017]. Moreover, from psychosocial factors both self-stigma [CI (−0.367, to 0.190), *p* < 0.001] and self-esteem [CI (−0.007, 0.033), *p* = 0.041] found to be the significant predictors of asthma control [*R*^2^ = 0.243, *F*_(2, 149)_ = 23.927, *p* = 0.001].

**Table 6:** Predictors of asthma control from socio-demographic and medical data (model 1), and self-stigma and self-esteem (model 2).

	**Unstandardized coefficients**			**95.0% Confidence interval for B**	
	**B**	**S.E**	***p***	**Lower bound**	**Upper bound**
^**a**^**Predictors Model 1**					
Constant	20.627	2.809	0.000	15.075	26.180
Gender	−1.043	0.820	0.206	−2.664	0.579
Age (years old)	−0.029	0.026	0.259	−0.080	0.022
Ethnicity	0.273	0.385	0.478	−0.487	1.034
Highest completed education level	−0.074	0.338	0.827	−0.742	0.594
Occupation	0.208	0.205	0.311	−0.196	0.612
Income per household/month (RM)	0.129	0.343	0.706	−0.548	0.807
Years since diagnosed as asthmatic	−0.007	0.025	0.788	−0.056	0.042
Emergency visits	−0.758	0.223	0.001^*^	−1.199	−0.317
Hospitalization	−0.304	0.487	0.534	−1.266	0.659
^**b**^**Predictors Model 2**					
Constant	29.603	4.058	0.000^*^	21.585	37.620
Self-stigma	−0.278	0.045	0.000^*^	−0.367	−0.190
Self-esteem	0.180	0.087	0.041^*^	0.007	0.353

a *Model 1*:

*Predictors: (Constant), Age, Gender, Occupation, Education level, Ethnicity, Income/ household/ month (RM), Years since diagnosed as asthmatic, Emergency visits, Hospitalization*.

*Dependent Variable: Asthma control*.

* *p < 0.05*.

b Model 2:

*Predictors: (Constant), Self-stigma, Self-esteem*.

*Dependent Variable: Asthma control*.

* *p < 0.05*.

## Discussion

Empirical research directly addressing self-stigma and self-esteem in asthma is sparse. One difficulty in studying this topic was the lack of appropriate measures. Therefore, a new questionnaire on self-stigma in adults was developed, pilot tested and utilized in the present study. This study aimed to assess the levels of self-stigma, self-esteem, and asthma control, and to investigate the impact of socio-demographic, medical and psychosocial factors on asthma control in Malaysian adults living with asthma. The enrolled patients showed moderate levels of self-stigma and self-esteem, and not well-controlled asthma.

In present study, statistically significant differences were recorded in stigma scores across BMI categories from all other independent variable categories of socio-demographic and medical data. The self-stigma score was the lowest in normal weight patients (59.82 ± 4.78) as compared to overweight and obese enrolled asthma patients. The higher stigma score in overweight and obese asthma patients might be because of the phenomenon of stigma layering as suggested by Lekas et al. (15). They found that the patients facing multiple sources of stigma were more prone to high level of stigmatization because of stigma layering. On contrary to the findings of present study, a previous study found that score of asthma-related stigma varied significantly in different age groups. The different findings of latter study might be because of the different targeted age groups (16). In Clark's study, the targeted sample was children and adolescent whereas this present study only enrolled the adult asthma patients.

Andrews et al. (5) found negative correlation between stigma and asthma morbidity. Some studies reported similar correlation of stigma that negatively influenced the control and health related outcomes in chronic diseases. Taft et al. (17) found negative correlation between stigma and control of irritable bowel syndrome i.e., greater levels of flare ups were linked with higher levels of perceived stigma. Moreover, self-stigma may also influence the control of the disease. Such detrimental impact of stigma in epileptic patients was studied by Smith et al. (18). They found that stigma was the significant barrier to the access of health services that in turn resulted into lack of control of disease symptoms. Few studies identified the underlined reasons of the negative correlation with the control of the disease. Sturdy et al. (19) suggested that “uncontrolled asthma may give rise to psychosocial or health behavior problems which reduce the quality of care and set into motion a vicious spiral of uncontrolled asthma interacting with psychosocial adversity.” Therefore, stigma was a common variable that affected the patients psychologically as well as physically and was associated with poor asthma control. Similarly, Kaptein et al. (20) observed that stigmatized and anxious patients were more prone to be less successful in coping and adapting to their illness and such patients could not achieve and maintain control of their illness.

The differences of self-esteem scores among different variable categories from socio-demographic and medical data were statistically insignificant. Similar to the present study, Cavusoglu (21) and McNelis et al. (22) found no statistically significant difference in self-esteem score in males and females. However, Harper and Marshall (23) and King et al. (12) reported that males had significantly higher self-esteem that females.

The global asthma report 2014 suggested that self-stigma of asthma somehow partly contributes to avoidance of inhaler in public and related with successful adaptation of the disease and subsequently the optimal control and minimized severity of asthma related morbidities (24). Moreover, stigmatization is considered as one of the major factors of intentional poor medication adherence as suggested in “*Treating to control symptoms and minimizing future risk”* section of GINA main report, 2019. Healthcare providers including pharmacists can deliver asthma education and training that is imperative to increase symptoms-free days and decrease healthcare utilization (2). One Malaysian-based qualitative study cited the feeling stigmatized to use inhaler in public as one of the major patient-related barriers to asthma management; therefore, to improve overall asthma management and health outcomes, it is critical to identify and address the barriers and challenges as perceived by asthmatic patients (25). In Malaysia, the Medication Therapy Adherence Clinics (MTAC) were initiated in 2009, which aimed to ensure the medication adherence by patient education and counseling across various chronic illnesses including asthma (26). The campaign was not targeted primarily at addressing psychosocial concerns like self-stigma and self-esteem *per se*; though recently some focus has been given to address patients' psychological issues of anxiety and depression but in an unstructured manner. In view of moderate level of self-stigma and self-esteem that influenced asthma control, the MTAC program can further be extended to improve patients' psychosocial well-beings.

This study is not without limitations. Several limitations should be considered in the evaluation of our findings. The cross-sectional design of this study prevents any conclusions about causal relationships between study variables and asthma control, which could potentially be evaluated in a longitudinal study. Second, the patients were enrolled in only one state of Malaysia: Selangor and included a disproportionately high number of women; consequently, our findings may not be generalizable to the broader population of adult asthmatic patients. Third, recall bias might also have affected the responses of patients to the questionnaires, which is a common limitation of surveys. Lastly, the proportions of variability in asthma control explained by the models were 12.9% (from variables of socio-demographic and medical data) and 24.3% (self-stigma and self-esteem). Although these values are not particularly high, it is not unusual to obtain lower values for *R*^2^ when predicting self-reported psychosocial factors. The demonstration of statistically significant relationships is of interest and potentially of clinical value. Such results present an opportunity for exploration in future research. Despite these limitations, our findings provide valuable real-world results. The study questionnaires were translated and validated (self-stigma and self-esteem) into the local Malay language to ensure a true manifestation of the Malaysian population.

After identifying the levels of self-stigma and self-esteem using this study instrument on scheduled day of appointment, the harmful consequences of high stigma and low self-esteem can be minimized by tailoring educational and behavioral interventions. Better campaigns to reduce self-stigma and improve asthma patients' self-esteem can only be designed and implemented by greater understanding of healthcare providers about asthma patient's perspectives at individual level. In order to reduce self-stigma of asthma, social support as well as support from healthcare teams may be helpful in tackling the negative outcomes of stigma of asthma. The peer support to the asthma patients may provide emotional as well as social support that in turn promote self-efficacy, self-esteem and positive health outcomes (19, 27). Reduced stigma and improved self-esteem of asthmatics can be ensured through encouragement, reinforcement, reassurance by healthcare professionals based on feedbacks from the patients by considering patients' psychosocial concerns and level of disease understandings (6, 28). This in turn may substantially influence the patient-provider relationship for better reflection of lived experiences of adults with asthma based on such concerns. The healthcare providers should translate these facts into practice while encapsulating social and psychological aspects of patient care. This may complement successful medical treatment of asthma patients. Furthermore, the supportive environment in terms of social/peer support and tailored self-management asthma education programs may contribute to improve the psychosocial well-being of the asthma patients. The results from this study provide rationale for future asthma-control investigations focusing on psychosocial issues. Addressing these areas has potential to improve asthma control, decrease patients' risk of exacerbations and overall quality of life.

## Data Availability Statement

All datasets generated for this study are included in the article/supplementary material.

## Ethics Statement

The studies involving human participants were reviewed and approved by Postgraduate Academic and Ethics Committee, UiTM (600-FF-(PT-9/19)), Research Management Institute, UiTM (600-RMI-(5/1/16)), Medical Research and Ethics Committee (MREC), Ministry of Health (MOH), Malaysia, through the National Medical Research Registry (NMRR-14-557-20184). The patients/participants provided their written informed consent to participate in this study.

## Author Contributions

SA and NI contributed conception and design of the study. SA, MZ, and AI collected the data from study settings. SA and NI performed the statistical analysis. SA wrote the first draft of the manuscript. SA, NI, MZ, and AI wrote sections of the manuscript. All authors contributed to manuscript revision, read, and approved the submitted version.

### Conflict of Interest

The authors declare that the research was conducted in the absence of any commercial or financial relationships that could be construed as a potential conflict of interest.
